# Epidemiology of adult trauma injuries in Malawi: results from a multisite trauma registry

**DOI:** 10.1186/s40621-022-00379-5

**Published:** 2022-04-19

**Authors:** Linda Chokotho, Kevin Croke, Meyhar Mohammed, Wakisa Mulwafu, Jonna Bertfelt, Saahil Karpe, Sveta Milusheva

**Affiliations:** 1 Department of Surgery, College of Medicine, University of Malawi, Mahatma Gandhi, Blantyre, Malawi; 2 Department of Global Health and Population, Harvard T.H. Chan School of Public Health, 677 Huntington Ave, Boston, MA USA; 3 Development Impact Evaluation, World Bank, 1818 H St NW, Washington, DC 20433 USA

**Keywords:** Trauma care, Trauma registries, Road traffic accidents, Malawi

## Abstract

**Background:**

Large-scale multisite trauma registries with broad geographic coverage in low-income countries are rare. This lack of systematic trauma data impedes effective policy responses.

**Methods:**

All patients presenting with trauma at 10 hospitals in Malawi from September 2018 to March 2020 were enrolled in a prospective registry. Using data from 49,241 cases, we analyze prevalence, causes, and distribution of trauma in adult patients, and timeliness of transport to health facilities and treatment.

**Results:**

Falls were the most common mechanism of injury overall, but road traffic crashes (RTCs) were the most common mechanism of serious injury, accounting for (48%) of trauma admissions. This pattern was consistent across all central and district hospitals, with only one hospital recording < 40% of admissions due to RTCs. 49% of RTC-linked trauma patients were not in motorized vehicles at the time of the crash. 84% of passengers in cars/trucks/buses and 48% of drivers of cars/trucks/buses from RTCs did not wear seatbelts, and 52% of motorcycle riders (driver and passenger) did not wear helmets. For all serious trauma cases (defined as requiring hospital admission), median time to hospital arrival was 5 h 20 min (IQR 1 h 20 min, 24 h). For serious trauma cases that presented on the same day that trauma occurred, median time to hospital arrival was 2 h (IQR 1 h, 11 h). Significant predictors of hospital admission include being involved in an RTC, age > 55, Glasgow Coma Score < 12, and presentation at hospital on a weekend.

**Conclusions:**

RTCs make up almost half of hospitalized trauma cases in this setting, are equally common in referral and district hospitals, and are an important predictor of injury severity. Pedestrians and cyclists are just as affected as those in vehicles. Many of those injured in vehicles do not take adequate safety precautions. Most trauma patients, including those with serious injuries, do not receive prompt medical attention. Greater attention to safety for both motorized and especially non-motorized road users, and more timely, higher quality emergency medical services, are important policy priorities for Malawi and other developing countries with high burdens of RTC trauma.

**Supplementary Information:**

The online version contains supplementary material available at 10.1186/s40621-022-00379-5.

## Background

The World Health Organization estimates that more than 5 million people die each year due to injuries, comprising 9% of global mortality, more than 1.5 times the number of deaths due to HIV/AIDS, tuberculosis, and malaria combined. More than 90% of these deaths occur in low- and middle-income countries (LMICs). This reflects both higher rates of injury in LMICs as well as weaker trauma care systems to treat injury (Reynolds et al. 2017). In addition, tens of millions of people each year suffer nonfatal injuries which require treatment and can result in ongoing health problems (World Health Organization 2014). For example, the WHO estimates that 10–15% of RTCs in low-income countries result in long-term disability (World Health Organization 2015). Sub-Saharan Africa in particular is facing a rapidly growing burden of trauma from injuries, which has been referred to as “Africa’s silent epidemic” (Orekunrin 2013).

Yet while injury rates in sub-Saharan Africa are among the highest in the world, the lack of comprehensive, reliable data on the epidemiology of injuries makes it difficult for policymakers to formulate effective policy responses. Prospective, registry-based data sources about injuries are scarce (Botchey et al. 2017; Juilliard et al. 2014; Croke et al. 2020). Even as the burden of trauma grows, health systems in sub-Saharan Africa are not comprehensively equipped to address and treat trauma. The absence of surveillance systems for identifying trauma cases and facilitating data-driven response, the lack of prehospital trauma care, and the reality of inaccessible and inadequate hospital care all contribute to the burden of preventable death, disability, and suffering from trauma (Chokotho et al. 2017; Mulwafu et al. 2017).

Malawi, a low-income country in southern Africa, faces a number of health-related challenges, including high rates of communicable disease, and elevated maternal and under-5 mortality, as well as a growing burden of noncommunicable diseases (NCDs) and injuries, which account for an estimated one-third of all death and disabilities (Ministry of Health 2018). Malawi exemplifies trends seen elsewhere in Africa: notably a rapidly growing trauma burden (Young et al. 2016) and a lagging policy response (Mulwafu et al. 2017). In Malawi, there are gaps in the trauma care system, but also in the data infrastructure which would allow policymakers to understand the scope of the problem. Trauma registries in Malawi to date have been largely based at referral hospitals (e.g., Queen Elizabeth Central Hospital, Kamuzu Central Hospital) with several more limited registries in single district hospitals over a limited period. However, data from these sites have not been pooled or collected prospectively as a multisite trauma registry. As a result, there has been no broad-based measurement of trauma across different regions of the country and in different social and economic contexts (i.e., across urban and rural areas, and in both referral and district hospitals). This means that broader trends with respect to trauma in Malawi, especially outside of the larger cities of Lilongwe and Blantyre, have not been comprehensively measured. This has limited the ability of policymakers to quantify the size of Malawi’s trauma problem, and hampered their efforts to develop, target, and evaluate policy interventions.

Several existing studies provide information on trauma in sub-Saharan Africa (see Table 1 for a summary of selected studies and their main findings), but two critical gaps arise in the literature. The first gap relates to the specifics of traumatic injuries and access to emergency care, such as the activity undertaken at the time the injury occurred, the location and nature of trauma injuries; time from injury until arrival in hospital, modes of transport to hospital, and the timeliness of treatment upon arrival at hospital. A second gap in the literature is that many studies have been based on a single facility, most often large referral hospitals. While referral hospitals see a significant amount of the trauma caseload in many settings, it remains unclear how representative such facilities are. In Malawi, as in most countries, referral hospitals are located in urban centers, yet 84% of the population in Malawi live in rural areas and many face significant financial barriers to travel to urban hospitals. Using a fuller set of variables, and including 10 facilities spanning central and district hospitals, and from both rural, peri-urban, and urban settings, the analysis in this paper helps to fill these gaps in the literature.

**Table 1:** Summary of trauma registry studies in Sub-Saharan Africa

Trauma indicators	Summary of existing evidence on trauma in Sub-Saharan Africa						
	Chichom-Mefire et al. (2017)	Botchey et al. (2017)	Kobusingye et al. (2002)	Nicol et al. (2014)	Samuel et al. (2009)	Chokotho et al. (2019)	Sawe et al. (2020)
Country	Cameroon	Kenya	Uganda	South Africa	Malawi	Malawi	Tanzania
Timeline	January 2008–October 2013	January 2014–May 2015	January 1998–December 1998	October 2010–September 2011	February 2008–June 2008	May 2013–May 2015	February 2019–September 2020
Number of trauma cases	5617	14,237	4359	9236	1474	3747	6302
Number of facilities	1	4	5	1	1	1	5
Causes of trauma							
RTC	55.1%	36.80%	50%	18.80%	43.40%	31.60%	60.3%
Falls	4%	26.40%	13%	18.40%	13.50%	10.00%	18.50%
Assault	21.8%	NA	NA	20.90%	24%	38.2%	5.40%
Stab/Cut	NA	8.20%	16%	NA	NA	NA	3.00%
Blunt force trauma	NA	NA	NA	17%	NA	NA	NA
Serious trauma							
Share of mild trauma	94%	NA	97%	NA	NA	NA	83%
Intent							
Share of intentional injuries	NA	NA	13%	32.10%	NA	33.40%	8.90%
Share of fractures	NA	NA	NA	NA	14.30%	13.00%	24.90%
Share of cases admitted	6.40%	24%*	33.00%	NA	26.80%	14.30%	44%*
Share of deaths	0.40%	2.40%	0.60%	NA	3.50%	0.10%	2.10%
Share of males	67%	76.10%	72.30%	71.30%	75.70%	79.10%	71.30%
Mean or Median age	26.8^+^	28^+^	24.2^+^	25–44^	< 5 and 26–30^#^	32^+^	27^~^

^*^Every trauma case where patient was admitted to ward, ICU or operation is included

+ Mean of age is reported

^Mode of age is reported

^#^Age distribution reported is bimodal

~ Median of age is reported

The objective of this study is to better understand the epidemiological patterns and care of injuries using multisite trauma registry data in Malawi, with a specific focus on causes of serious trauma which require hospital admission. We document the main mechanisms of injury and types of injuries, the patterns of care seeking and referral, and the patterns of treatment for trauma. This study contributes to the literature on trauma in developing countries by analyzing patterns of trauma injuries and trauma care from one of the largest multisite comprehensive trauma registry datasets for sub-Saharan Africa.

## Methods

**Fig. 1:**
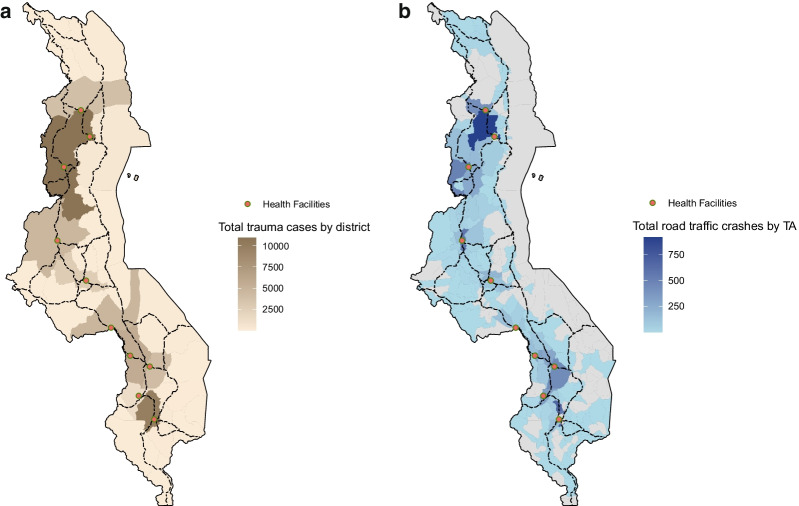
Total number of trauma cases and crashes by location. **a** (Left) Total number of trauma cases by district. **b** (Right) total number of RTC by Traditional Authority (TA). The panel shows total number of trauma cases by district (**a**) with 48,747 trauma cases (1% cases were missing information on district); and the total number of RTC by Traditional Authority (TA) (**b**) for 8565 RTC cases (7% of RTC cases were missing or had incomplete information on TA in the trauma registry.)

Details of the data collection process are published in Croke et al. (2020). The analysis in this paper is based on data collected in 10 health facilities (two central hospitals, seven district hospitals, and one community hospital) as part of a collaboration between the World Bank and Malawi’s Ministry of Health. Figure 1 shows the geographic scope of the trauma registry and the frequency of cases recorded in the registry by district (a) and subdistricts (b) over the data collection period. The trauma registry comprised seven district facilities (1/3rd of Malawi’s district hospitals), two out of Malawi’s four referral hospitals, and one community hospital. The facilities are located along the main highway in the country (the M1) and span across most of the north–south length of the country (Fig. 1). The trauma registry contains data on 118,013 trauma cases from August 2018 to June 2021. This paper presents data from September 2018 to March 2020, excluding the first month of data collection (a pilot phase of data collection), as well as data after April 2020, when the COVID-19 pandemic began to affect health system utilization (42,717 cases excluded).

In the registry, trauma patients were defined as those who had sustained one or multiple injuries to any body region or regions within the last 30 days. Analysis focuses on patients above the age of 15, since pediatric trauma cases were handled differently and data were recorded using different methods in several facilities (this excludes 26,017 trauma cases for individuals under age 15). Cleaning of the data included incorporating these exclusion conditions and removing all duplicate patients and removing any other cases (38) collected from Queen Elizabeth Central Hospital’s (QECH) Accident and Emergency (A&E) unit (because this unit is focused on pediatric cases). After incorporating all these restrictions, 49,241 cases remain in the study sample from September 2018 to March 2020. Data analysis is conducted for the following categories of variables collected in the trauma registry: demographic information, mode of transport to hospital, geographic location of trauma, time of trauma, time of hospital arrival and time attended, setting, intent and cause of trauma, vital signs, AVPU (alert, voice, pain, unresponsive) scale, Glasgow Coma Score, and diagnosis.

We distinguish between the seriousness of trauma cases on the basis of whether patients were hospitalized to the facility or else treated and discharged the same day, as well as based on GCS and APVU scores and patient reports of pain intensity. We also calculate summary trauma scores (the Kampala Trauma Score). The study presents descriptive statistics that characterize trauma patients, the care they receive, and the outcome of their hospital visit. In the appendix (Additional file 1: Table S2), multivariate logistic regression models are used to analyze associations between patient and injury characteristics and inpatient admission.

## Results

This section presents trends observed and analysis for 49,241 trauma cases collected between September 2018 and March 2020 across 10 health facilities in Malawi.

### Demographic information

**Fig. 2:**
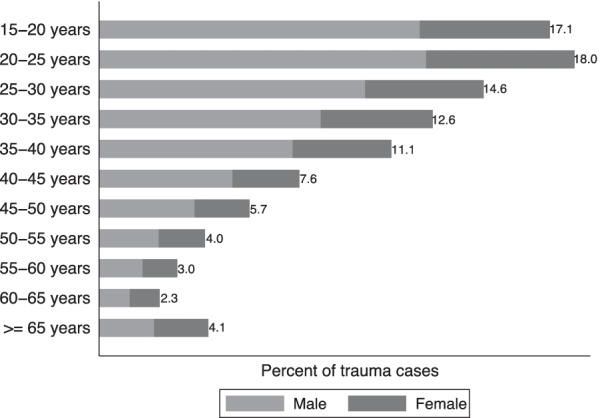
Age and gender. The graph shows the age groups on the vertical axis and the percent of trauma cases from each age group on the horizontal axis

Figure 2 shows the demographic correlates of trauma cases in the sample. Injuries are most common for younger adults (median age of 30; IQR 22, 40). Two-thirds of trauma patients are male, with greater gender differences at younger ages. Overall, 33% of all trauma cases were females and 66% males. For serious injuries (i.e., AVPU < 4, GCS < 8, patients whose self-reported pain level was severe or extreme, or hospitalizations), 30% were females, and 60% were males (*p*-value < 0.001). Among hospitalized patients, 27% were females, and 73% were males (*p*-value < 0.001).

### Injury details

We examine mechanism of injury, type of injury, disposition, injury severity, and timeliness of care for non-hospitalized and hospitalized trauma cases, using hospital admission as a proxy for severity.

### Mechanism, diagnosis, and location of injury

**Fig. 3:**
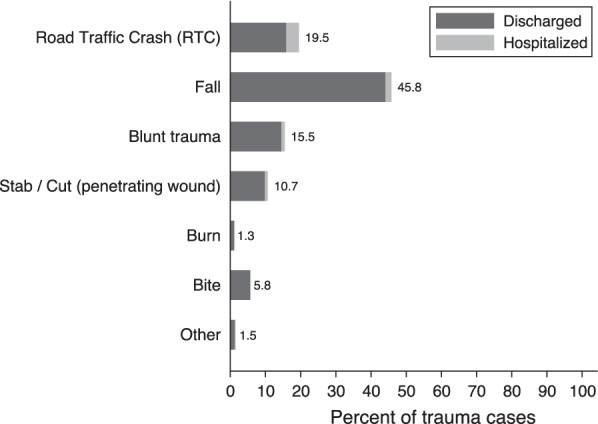
Mechanism of trauma injury. The figure represents the different mechanisms of trauma injuries as observed in the trauma registry data on the vertical axis and their percentage in the total trauma cases on the horizontal axis

**Fig. 4:**
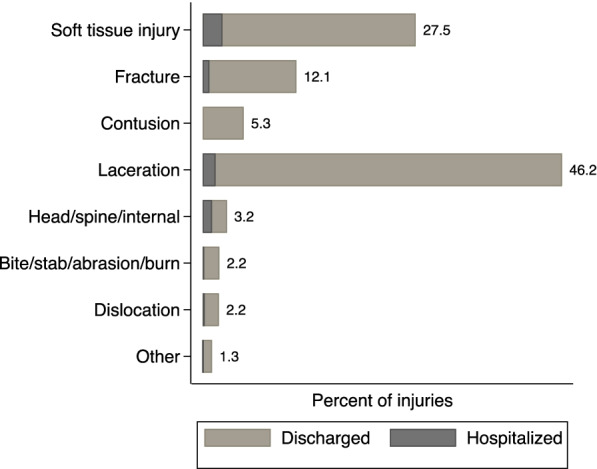
Type of injuries. The figure represents the type of injuries recorded in the trauma registry data on the vertical axis and the percentage of those injuries on the horizontal axis

The most common mechanisms of injury are falls (45.8%), followed by RTCs (19.5%), blunt trauma (15.5%), stabs and cuts (10.7%), and bites (5.8%) (Fig. 3). The most common diagnosis across all trauma cases is soft tissue injuries and contusions (46%), followed by fractures (27%), lacerations (12%), bites (5%), penetrating wounds (2%), burns (1%), and dislocations (2%) (Fig. 4). For hospitalized trauma cases, the most common mechanism of injury was RTCs (48%), falls (22%), blunt trauma (12%), and penetrating wounds (11%). The most common type of injuries for hospitalized patients was fracture (35%), followed by soft tissue injuries and contusions (23%), and lacerations (12%). For all trauma cases, 71% of injuries were to the extremities, while 7% were to head and neck, and 8% to the face, 3% to the thorax, and 2% to the abdomen.

### Injury severity

Injury severity (proxied by hospital admission) varied by injury cause. Hospital admissions include all cases where a patient was admitted to a ward, intensive care unit (ICU), operating theatre, or if the patient died in the casualty department. While falls were the most common mechanism of injury, only 4% resulted in admission. By contrast 18% of RTCs resulted in admission. Overall, 7% of trauma patients were hospitalized (admitted overnight). 21% (768) of hospitalized cases had injuries in the head and neck region. In addition to hospital admission, other measures of injury severity recorded in the trauma registry are Glasgow Coma Scores (GCS) and AVPU score, as well as the patients’ subjective pain rating. Out of all hospital admissions with injuries in the head and neck region (768), 11% had severe injuries (GCS ≤ 8), 11% had moderate injuries (GCS between 9 and 11). Overall across all trauma cases, 40% of patients reported none or mild pain, 53% reported moderate pain, and 6% either severe or extreme pain. 4% of patients had a Kampala Trauma Score lower than 14, denoting moderate to severe injury.

### Variation across health facilities

**Fig. 5:**
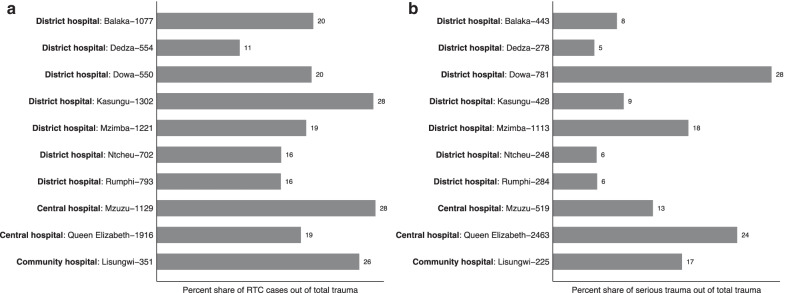
Percentage of RTC and serious cases out of total trauma. **a** (Left): Percentage of RTC out of total trauma. **b** (Right): Percentage of serious cases out of total trauma. The panel shows (**a**) the percentage of RTC out of total trauma for each hospital. The number next to the hospital name represents the number of RTC cases in the hospital. **b** shows the percentage of the serious trauma cases out of total cases for all hospitals. The number next to the hospital name represents the number of serious cases in the hospital. Serious trauma is defined as trauma cases where patients require hospital admission, or GCS < 8, or AVPU < 4, or subjective pain level is severe or extreme

A unique feature of this registry is the broad coverage of multiple levels of health facilities: referral hospitals (2) and district (7) and community hospitals (1). We analyze the distribution of trauma across these categories of hospitals. Facilities varied significantly in the number of trauma cases seen, percentage of patients hospitalized, the percentage of patients who present with serious trauma, and the percentage of trauma caused by RTCs (Fig. 5). RTC cases as percent of the total trauma caseload varies within a narrow range (16–28%) with the exception of Dedza District Hospital; this hospital is 1.5 h from Kamuzu Central Hospital and other facilities in Malawi’s capital, Lilongwe, so RTCs nearer to Lilongwe than Dedza may divert to these facilities. Hospitals vary widely in the percentage of cases admitted (from 3 to 16%) and in the percentage of patients presenting with serious trauma, as defined in the previous section (from 5 to 28%), with referral hospitals seeing approximately two times as much serious trauma, as a percentage of total caseloads, as district hospitals (21% to 11%).

### Patterns of RTCs

Since RTCs represent almost half of all trauma cases requiring hospital admission, we examine them in more detail here. We report information on road users, vehicles involved, and crash details. 1.3% (133/9,595) of RTC patients were dead on arrival at the facility and 18% required admission to hospital. Soft tissue injuries and contusions are observed in 56% of RTC patients, followed by fractures (20%), and lacerations (11%). RTCs make up 21% of all trauma cases in central hospitals, compared to 19% for district hospitals. Additional file 1: Table S1 shows the detailed breakdown of the number of trauma cases, RTCs, and admission by hospital.

**Fig. 6:**
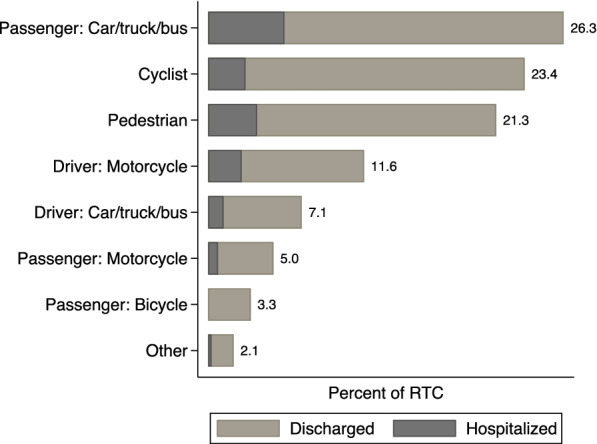
Role on the road for RTC injuries. The figure represents the types of road users on the vertical axis and their percentage in the trauma registry’s road traffic crashes on the horizontal axis

**Fig. 7:**
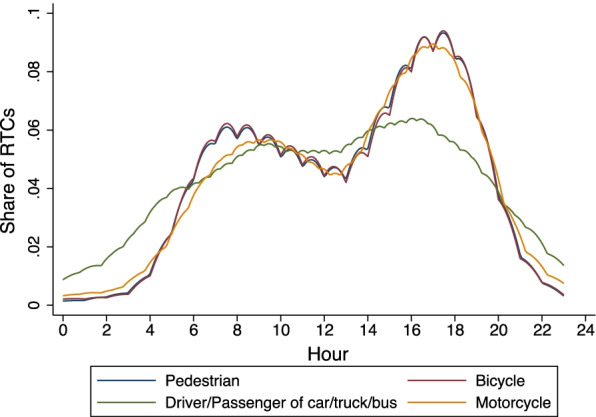
Distribution of RTC by road users and time of day. In the figure, each line represents a road user recorded in the trauma registry. The figure represents the percentage of road traffic crashes recorded in hour of a 24-h day (on the horizontal axis) for each road user

Non-motorized road users (pedestrians, cyclists, cart users) make up approximately half of all RTC trauma patients (49%) (Fig. 6). Among hospitalized road traffic crash patients, passengers of car/bus/trucks make up a third (32%) of the road users in RTC cases, followed by pedestrians (20%) and cyclists (16%). Overall, 50% of hospitalized pedestrians were struck by private vehicles or trucks, 23% by public transit vehicles, 17% by motorcycles, and 4% by bicycles. We present geographic variation in pedestrian crashes in the regions surrounding the facilities by looking at the percent of RTCs involving pedestrians out of the total RTC recorded in each facility from the 10 hospitals. Queen Elizabeth Central Hospital (45%), Dedza District Hospital (20%), Balaka District Hospital (18%), Ntcheu District Hospital (17%), Mzuzu Central Hospital (17%), and Kasungu District Hospital (16%) each have more than 15% of the RTC caseload involving pedestrians (see Additional file 1: Table S1 for total number of RTC for each hospital). Figure 7 shows the peak hours of road traffic crashes as recorded in the trauma registry. There are two peaks of RTCs consistent across all the road users coinciding with morning and evening rush hours, one between 4:00 and 8:00 h, and a second peak between 16:00 and 20:00 h. Non-motorized users (pedestrians and cyclists) have a higher percentage of RTCs happen during those peaks and almost none in the night hours. By contrast cars, trucks, and buses have a higher percentage of crashes between 23:00 and 4:00 h, likely due to lower visibility, fatigue, or speeding, and the reduced presence of pedestrians on roads. Presence of alcohol was noted, based on self-report, in 6.7% (45/678) of all drivers of cars, trucks, buses and suspected for 2% (15/678).

### Hospital care and trauma outcome

Median time elapsed between occurrence of trauma and patient arrival at hospital is 8 h 59 min (IQR 1 h 50 min, 23 h 50 min). However, this aggregate figure includes minor cases for which rapid treatment is not needed. We separately examine timeliness of treatment for the subset of severe trauma cases that represent urgent need for care based on four characteristics: patients who were admitted to hospital, or with AVPU < 4, or with GCS < 8, or patients whose self-reported pain level was severe or extreme. Finally, long recorded delays can also reflect patients’ decisions not to seek care immediately, and therefore may not reflect gaps in emergency transport. Therefore, we also examine time elapsed for the subset of patients who seek care on the same day (within 24 h of the trauma).

**Fig. 8:**
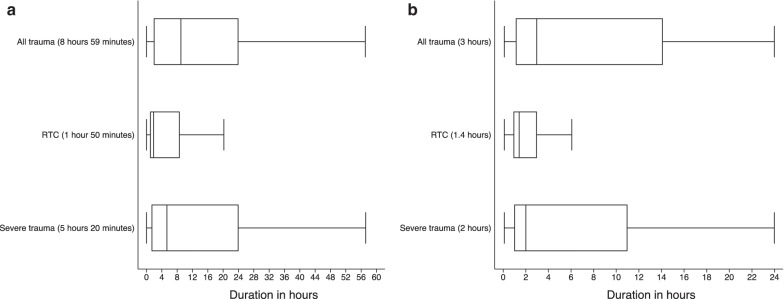
Time to arrival. **a** Time to arrival for all trauma cases. **b** Time to arrival for cases that arrived on same day as trauma. The figure shows the distribution of duration of arrival to the facility since trauma for all cases (on the left) and for cases that arrive at the facility on the same day as the trauma (right). The vertical axis represents the distribution of arrival for all trauma, road traffic crashes and all severe trauma. The horizontal axis represents the duration times in hours. The line inside each box represents the median duration of arrival (also in parentheses). All referred trauma cases are excluded

**Fig. 9:**
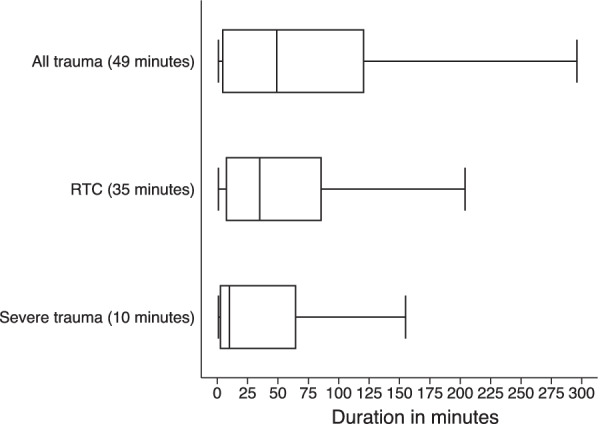
Time to receive care after arrival for all trauma. The figure shows the distribution of duration to get care after arrival to the facility in minutes. The vertical axis represents the distribution for all trauma, road traffic crashes and all severe trauma. Referrals are excluded

Figure 8 shows the arrival times and time to receive care post-arrival across all trauma, RTCs, and severe trauma. Severely injured patients arrive after a median time of 5 h 20 min (IQR 1 h 20 min, 24 h), and RTC patients arrive after a median time of 1 h 50 min (IQR 57 min, 8 h 40 min). For seriously injured patients who seek care on the same day as their trauma, median time elapsed between occurrence of trauma and patient arrival at hospital is 2 h (IQR 1, 10 h). However there is notable variation across hospitals (Additional file 1: Fig. S1), and for non-RTC serious trauma, the median time increases to 4 h. For RTCs, patients are seen by a clinician within a median 35 min after arrival. For minor trauma, patients are seen approximately an hour after their arrival. Severely injured patients are seen within 10 min of their arrival (Fig. 9).

For hospitalized trauma cases, the most common modes of transport to hospitals are private (29%) and commercial vehicles such as taxis (21%), public modes such as minibuses (16%), and ambulances (6%).

### Disposition

The trauma registry also records the final outcome of the trauma case in the casualty department on the day they visit the hospital. 92% (45,374) of all trauma cases were treated and sent home the same day, 6.5% (3,232) of cases were admitted to another ward, 0.1% (74) were taken to the operating theatre, 0.04% (19) were taken to the ICU, 0.1% (49) of patients died in the casualty department, 0.5% (242) were referred to another facility, and 0.4% (177) were dead on arrival. Of all patients admitted in the ward, intensive care unit, or operating theatre, 1,636 (49%) patients remained in the hospital 24 h later. Information about the treatment provided was collected for patients who stayed in the facility overnight. For 88% of the cases that stayed overnight, a form of pain relief (diclofenac, Panado, paracetamol) was given, 32% of the cases were given antibiotics, 24% of cases received a plaster of Paris cast/backslab, and 21% of cases received blood, intravenous fluids, or oxygen. (If multiple treatments were given to a patient, all the treatments were recorded in the trauma registry.)

## Discussion

It is widely understood that trauma is a growing problem in many sub-Saharan African countries. However, outside of a group of trauma registries based primarily in large referral hospitals, systematic data collection on the burden of injuries in Africa has been lacking. This paper seeks to fill this gap with larger-scale data collection from both central and district hospitals in Malawi. These data show both similarities with trauma registries from other African settings as well as key areas of divergence. Furthermore, the broad scale of data collection demonstrates patterns which could be overlooked in single site registries or multiple site registries focused only on referral hospitals.

Compared to previous registries, the percentage of injuries stemming from falls is higher and the percentage from RTCs (19.6%) is lower. For example, the corresponding RTC rates in other sub-Saharan Africa trauma registries were 55% (Chichom-Mefire et al. 2017; Cameroon); 36% (Botchey et al. 2017; Kenya); 50% (Kobusingye et al. 2002; Uganda); and 43% (Samuel et al. 2009; Malawi). These trauma registries were largely implemented in tertiary or other urban referral hospitals. By contrast, in this registry, which includes more district hospitals located outside of urban areas, the most common mechanism of injury is falls. However, even in this setting, where minor trauma dominates the overall case load, we still find that RTCs make up almost half of hospitalized trauma cases (48%), and this fraction does not vary dramatically from central to district hospitals.

The less urban settings of many of the district hospitals in this registry may account for the differing nature of the RTC caseload. In several other settings, motorcycle-related crashes dominated, while in this registry, roughly half (49%) of RTC victims are non-motorized road users such as pedestrians and cyclists. This suggests the need for targeted policies and infrastructure to improve road safety for these users. These findings are consistent with those of Banza et al. (2016), who also found a heavy burden of injury from RTCs on pedestrians and cyclists in a Kamuzu Central Hospital trauma registry.

Like other registries, we also find limited adherence to safety practices for motorized RTCs, implying potential scope to reduce the burden of serious trauma. 84% of patients who were passengers of motor vehicles such as cars, buses, and trucks report not having worn a seat belt. While helmets are required by law for both drivers and passengers of motorbikes, only 45% of patients who were drivers of motorbikes report wearing a helmet. These findings are similar to those of Sundet et al. (2021) who find limited seat belt use among RTC patients in Lilongwe, Malawi. This highlights the scope for increased seat belt and helmet use to reduce RTC-related trauma. Malawi already has a motorcycle helmet law that applies to both drivers and passengers, and it also has a seat belt law that applies to front seat passengers (WHO 2018). Ngwira et al. (2020) estimate that seat belts are available in over 90% of motor vehicle; therefore, a lack of equipment is not the primary challenge. Instead, the WHO rates the enforcement of helmet use as 2 out of 10 and enforcement of seat belts as 3 out of 10 (WHO 2018). This suggests that evidence-based efforts to improve enforcement of laws or otherwise increase seatbelt and helmet usage should be developed and evaluated.

 This registry also shows that it cannot be assumed that serious trauma cases present to referral hospitals. While referral hospitals do see more serious trauma cases as a percent of total caseload, we also observe significant numbers of serious trauma cases at district hospitals. For the largest cause of trauma, RTCs, the largest aggregate caseload is a national referral hospital (QECH), but several district hospitals also faced caseloads, both in aggregate and as a percentage of their total trauma caseload, larger than the second referral hospital in our sample (Mzuzu Central Hospital). This highlights the need to improve the staffing and equipment of district hospitals to handle RTC and other serious trauma cases.

Another concerning finding is the major delays observed in transport to hospitals and treatment, which can be analyzed using the “three delays” framework (delays in the decision to seek care, delay from injury to hospital, and delay from arrival to being seen) (Calvello et al. 2015). Patients in this registry report long delays in time elapsed between injury and arrival at hospital (which reflect both delayed decisions to seek care and delays in transport), and delays in care after arrival in hospital. For all cases, the median time is 10 h. These delays are smaller for those who seek care on the same day, suggesting that many of the long recorded delays in care are due to delayed decision to seek care, in addition to delays in access to emergency transport. For cases that required admission (presumably more serious injuries with less delay in the decision to seek care), median time to hospital is 3 h, and for RTCs, median time is 2 h.

However, the role that lack of access to transport access plays is demonstrated by the fact that the location of the accident and access to transport is more closely linked to timeliness of care than injury severity: RTCs (both serious and non-serious), which happen on the road, where transport options are present, have a median time to arrival of just under 2 h (110 min). By contrast, serious non-RTC cases have a median time of 4 h. This comparison suggests major barriers to transport for non-RTC serious trauma cases. Furthermore only 6% of admitted (hospitalized) trauma cases use ambulances to reach hospitals. In this setting, ambulance transport is used more often for referral across facilities than for emergency transport from the trauma site to hospitals. Several other registries in the region have recorded much shorter prehospital delays (Chichom-Mefire et al. 2017; Kobusginye et al. 2002; Botchey et al. 2017). However these long delays across Malawi are consistent with findings from the Kamuzu Central Hospital registry in Lilongwe (Samuels et al. 2009). Further research is needed on the extent to which prehospital delays relate to the decision to seek care versus the availability and affordability of emergency transport.

A limitation of this study is that we do not have detailed data about quality of care received by trauma patients. A second limitation is that we only observe the population of patients who present to hospital. As a result, we cannot make inferences about trauma incidence at population level. Finally, we do not have data on health status of trauma patients after discharge. Understanding the health, economic and social well-being of trauma patients, including those suffering serious injuries from RTCs and other causes, over extended periods of time in this setting would be very valuable.

## Conclusions

This paper reports on the patterns of trauma injuries and trauma care in a multisite registry in Malawi, highlighting the correlates of injury and hospital admission, and documenting important gaps in timeliness of care. We highlight the importance of RTCs, which comprise about half of the trauma cases that require hospital admission, and are equally common in referral and district hospitals. We find that pedestrians and cyclists are just as affected as those in vehicles. We also note that many of those injured in vehicles do not take adequate safety precautions. Our findings also demonstrate that many trauma patients, including those with serious injuries, do not receive prompt medical attention. This analysis demonstrates the potential of trauma registries to inform both preventive policies and clinical care. Further development of registries, including with greater detail on care delivered and patient outcomes, could greatly contribute to the evidence base regarding trauma care in low-income settings.

## Supplementary Information

**Additional file 1**. **Figure S1**. Time to arrival for severe trauma cases that arrive within 24 hours of occurrence of trauma by hospital. **Table S1**. Hospital-level trauma cases. **Table S2**. Regression analysis: predictors of admission to hospital.

## Data Availability

Data are available from the authors upon reasonable request.

## Abbreviations

A&E: Accident and emergency
AVPU scale: Alert, verbal, pain, unresponsive scale
GCS: Glasgow come score
IQR: Interquartile range
KTS: Kampala trauma score
LMICs: Low- and middle-income countries
NCDs: Noncommunicable diseases
QECH: Queen Elizabeth Central Hospital
RTC: Road traffic crash
