# Supplementary material for: Epidemiology of adult trauma injuries in Malawi: results from a multisite trauma registry
Source: Inj Epidemiol. 2022 Apr 19;9:14. doi: 10.1186/s40621-022-00379-5 (PMC9017418; doi:10.1186/s40621-022-00379-5)

**Additional file 1: Appendix**

**Appendix Figures**

Additional file 1: Figure S1. Time to arrival for severe trauma cases that arrive within 24 hours of occurrence of trauma by hospital

|  |
| --- |
|  |
| **Note:** The above figure shows the distribution of duration of arrival to the facility since trauma for severe trauma cases that arrive at the facility on the same day as the trauma by each hospital. Severe trauma is defined as patients who were hospitalized, or with AVPU < 4, or with GCS < 8, or patients whose self-reported pain level was severe or extreme. The vertical axis represents the distribution of arrival for all trauma for each hospital in the trauma registry. The horizontal axis represents the duration times in hours. The line inside each box represents the median duration of arrival (also in parentheses). All referred trauma cases are excluded. |

**Appendix Tables**

**Additional file 1: Table S1. Hospital level trauma cases**


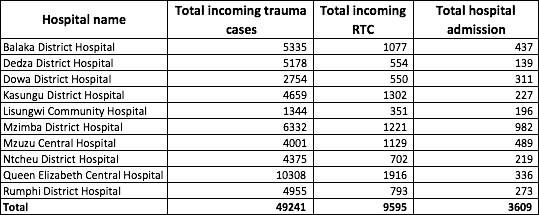


**Appendix: Statistical analysis**

In the regression models, we estimate the likelihood o patient $i$ outcome of admission $\left( Admission_{i} \right)$to a hospital using three models:

- **Demographic model**: This model uses only the demographic characteristics of a patient $i$, such as age $(Age_{i}),$ education level $(Education_{i}),$ gender $(Gender_{i})$and their occupation $(Occupation_{i})$ to estimate their likelihood of both outcomes.

$$Admission_{i}=\alpha+\beta_{1}Age_{i}+\beta_{2}Education_{i}+\beta_{3}Gender_{i}+\beta_{4}Occupation_{i}$$

- **Clinical model**: Under this model, we use demographics and adjust for clinical aspects of the injury to estimate predictors of admission and death. Additional variables include the type of injury suffered by patient$i$ ($Injury_{i}$), whether the trauma was RTC $(RTC_{i})$, whether the patient presented to a central or district hospital $(Central_{i})$, whether the injury happened during a weekend $(Weekend_{i})$, and whether it happened during daytime hours $\left( Day_{i} \right).$

$$Admission_{i}=\alpha+\beta_{1}Age_{i}+ \beta_{2}Education_{i}+ \beta_{3}Gender_{i}+ B_{4}Occupation_{i}+\boldsymbol{\beta}_{\boldsymbol{5}}\boldsymbol{Injur}\boldsymbol{y}_{\boldsymbol{i}}\boldsymbol{+}\boldsymbol{\beta}_{\boldsymbol{6}}\boldsymbol{RT}\boldsymbol{C}_{\boldsymbol{i}}\boldsymbol{+}\boldsymbol{\beta}_{\boldsymbol{7}}\boldsymbol{Centra}\boldsymbol{l}_{\boldsymbol{i}}\boldsymbol{+}\boldsymbol{\beta}_{\boldsymbol{8}}\boldsymbol{Weeken}\boldsymbol{d}_{\boldsymbol{i}}\boldsymbol{+}\boldsymbol{\beta}_{\boldsymbol{9}}\boldsymbol{Da}\boldsymbol{y}_{\boldsymbol{i}}$$

- **Clinical model with severe injuries**: In addition to clinical predictors used in the above model, in this model we also include variables capturing aspects of injury severity, in order to better predict hospital admission. Severe injuries are defined as injuries where the GCS score of the patient is below 12 $(GCS_{i});$ many of these are head injuries.

$$Admission_{i}=\alpha+\beta_{1}Age_{i}+ \beta_{2}Education_{i}+ \beta_{3}Gender_{i}+ B_{4}Occupation_{i}+\beta_{5}Injury_{i}+ \beta_{6}RTC_{i}+ \beta_{7}Central_{i}+ \beta_{8}Weekend_{i}+\beta_{9}Day_{i}+\boldsymbol{\beta}_{\boldsymbol{10}}\boldsymbol{GC}\boldsymbol{S}_{\boldsymbol{i}}$$

**Additional file 1: Table S2. Regression analysis: predictors of admission to hospital**


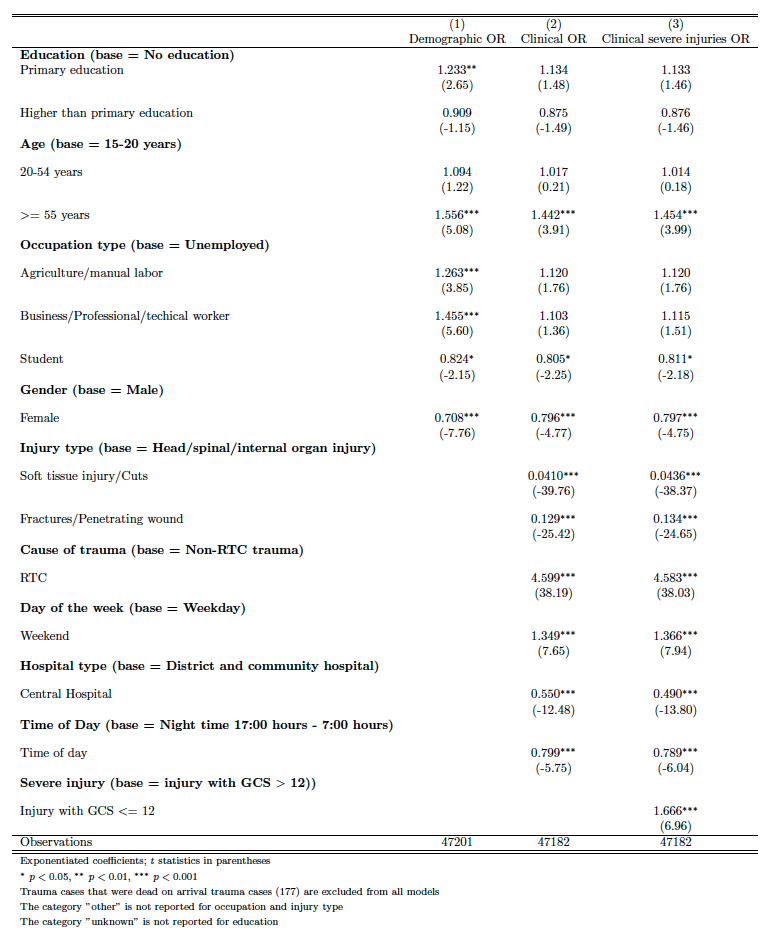

Supplement: Supplementary file 1 — Additional file 1. Figure S1. Time to arrival for severe trauma cases that arrive within 24 hours of occurrence of trauma by hospital. Table S1. Hospital-level trauma cases. Table S2. Regression analysis: predictors of admission to hospital. [file 40621_2022_379_MOESM1_ESM.docx]
